# Transgradient Variant of Mal De Meleda Presenting As Palmoplantar Keratoderma: A Case Report

**DOI:** 10.7759/cureus.60717

**Published:** 2024-05-20

**Authors:** Arshiya Khan, Adarshlata Singh, Bhushan Madke, Kunal Gupta, Talasila Sree Ramya

**Affiliations:** 1 Department of Dermatology, Jawaharlal Nehru Medical College, Datta Meghe Institute of Higher Education and Research, Wardha, IND

**Keywords:** transgradient variant, yellowish skin, acitretin, palmoplantar keratoderma, mal de meleda

## Abstract

Mal De Meleda is a rare genetic disorder characterized by palmoplantar keratoderma, often presenting challenges in diagnosis and management. This case report discusses an 18-year-old male presenting with thickened, yellowish skin on both palms and soles, accompanied by itching and cracking. A diagnosis of the transgradiens variant of Mal De Meleda was established through clinical and histopathological examination. Treatment with oral acitretin and topical moisturizers resulted in significant improvement. This report highlights the importance of recognizing rare variants of palmoplantar keratoderma and the need for a multidisciplinary approach to diagnosis and management.

## Introduction

Mal De Meleda is a rare autosomal recessive genodermatosis characterized by diffuse palmoplantar hyperkeratosis, usually without associated systemic manifestations [1]. It was first described by Stulli in 1826 and later by Meleda in 1829, hence its name [2]. The condition typically presents during infancy or early childhood, although cases with later onset have also been reported [3].

The transgradient variant of Mal De Meleda represents an exceedingly rare subtype, distinguished by a unique pattern of progression from the distal to proximal aspects of the extremities [4]. This variant poses diagnostic challenges due to its rarity and resemblance to other forms of palmoplantar keratoderma (PPK), such as Unna-Thost and Vorner [5]. Differential diagnosis may include other genodermatoses with palmoplantar involvement, such as Papillon-Lefèvre syndrome and keratoderma hereditarium mutilans [6]. Histopathologically, Mal De Meleda is characterized by hyperkeratosis, hypergranulosis, and acanthosis, with a pronounced stratum corneum thickening [7]. Molecular studies have identified mutations in the aminoacyl-tRNA synthetases (component B) gene on chromosome 8q24.3, responsible for encoding secreted mammalian Ly-6/urokinase-type plasminogen activator receptor-related protein-1, as the underlying genetic defect in Mal De Meleda [8].

Management of Mal De Meleda remains challenging, with limited treatment options providing only symptomatic relief. Oral retinoids, such as acitretin, have effectively reduced hyperkeratosis and improved symptoms [9]. However, long-term use may be associated with adverse effects, necessitating careful monitoring [10]. This case report contributes to the existing literature by presenting a rare case of the transgradient variant of Mal De Meleda, emphasizing the importance of recognizing atypical presentations and implementing appropriate diagnostic and therapeutic strategies.

## Case presentation

**Figure 1:**
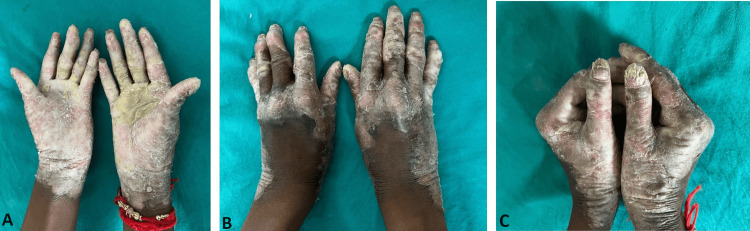
Transgradient PPK with sharp margins (A), conical tapering of the fingers (B), and loss of dermatoglyphics (C). Fingernails show thickening, longitudinal ridges, and dystrophic changes PPK: palmoplantar keratoderma

An 18-year-old male presented with yellowish, thickened skin on both palms and soles associated with multiple cracks and itching. The patient appeared healthy 18 years ago when he initially developed lesions on his soles, which later progressed to involve his palms over two years. The onset of symptoms was sudden, followed by gradual progression. The lesions were accompanied by mild itching, flaking, and cracking (Figure 1).

The patient reported itching, scaling, and a foul smell from the lesions, along with a history of previous local applications and no prior trauma. Treatment history revealed unsuccessful attempts with emollients, topical steroids, and salicylic acid ointments from a local hospital before seeking care at the dermatology outpatient department due to exacerbation. The patient's younger brother also exhibited similar complaints.

**Figure 2:**
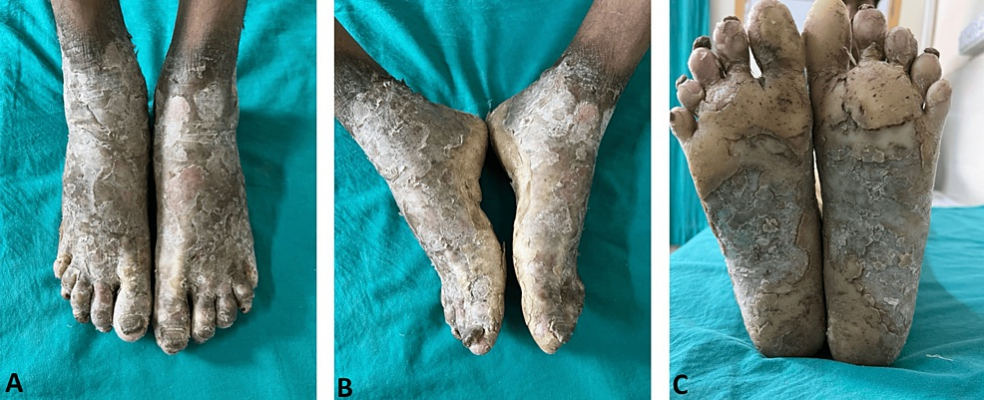
(A-C) Transgradient straw-yellow plantar keratoderma involving the soles and toes of the foot with well-defined margins

Upon examination, multiple yellowish waxy hyperkeratotic plaques with severe scaling and fissuring were observed on both palms and soles, extending to the wrists and ankles, with conical tapering of the distal digits. Additionally, the nails were affected, showing thickening, longitudinal ridges, and dystrophic changes (Figure 2).

**Figure 3:**
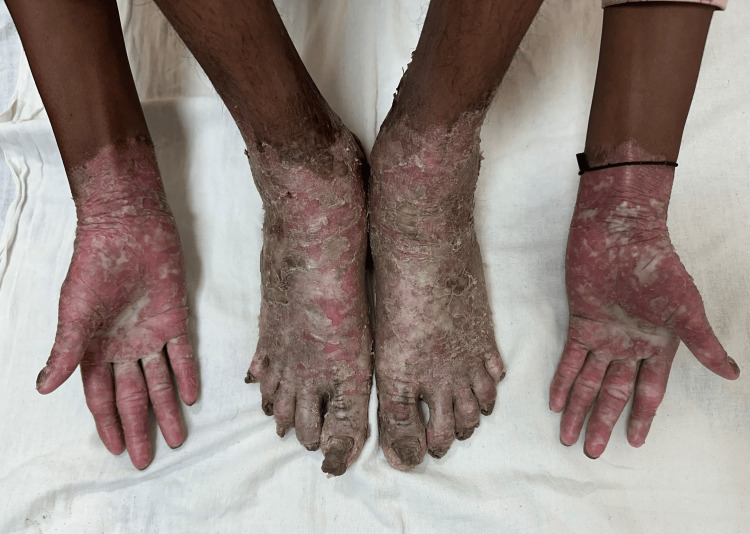
Multiple yellowish waxy hyperkeratotic plaques with severe scaling and fissuring observed on both palms and soles, extending to the wrists and ankles, with conical tapering of the distal digits

A diagnosis of the transgradient type of the Mal De Meleda variant of PPK was established. Radiographic evaluation of bilateral hands, wrists, and feet with ankles revealed no abnormalities. Histopathological examination of the skin lesions demonstrated hyperkeratosis, hypergranulosis, and acanthosis (Figure 3).

**Figure 4:**
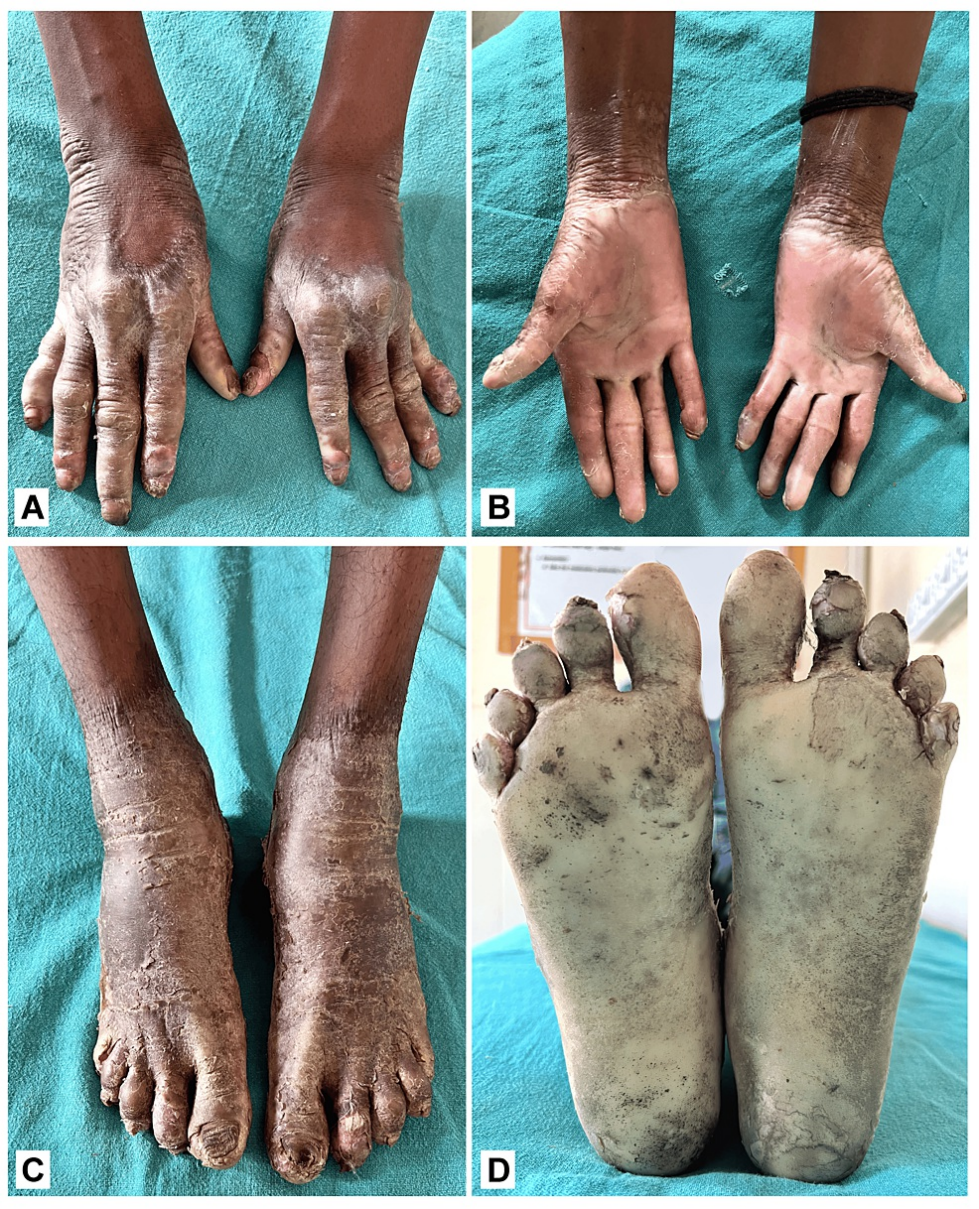
(A-D) Follow-up figures of the patient

Treatment commenced with oral acitretin 25 mg at bedtime and twice-daily application of topical urea-based moisturizers. After two months, the patient showed significant improvement and continues to receive ongoing care (Figure 4).

## Discussion

The transgradient variant of Mal De Meleda is an exceptionally rare subtype of PPK characterized by its distinct clinical and histopathological features [11]. PPK encompasses a heterogeneous group of disorders characterized by excessive skin thickening on the palms and soles. Mal De Meleda is one such subtype, typically presenting with diffuse palmoplantar hyperkeratosis without systemic involvement [9]. The transgradient variant is distinguished by its unique pattern of progression, extending from the distal to proximal aspects of the extremities, which sets it apart from other forms of PPK [3].

Diagnosing transgradient Mal De Meleda can be challenging due to its rarity and similarity to other forms of PPK. Clinical suspicion coupled with histopathological examination is crucial for accurate diagnosis. Histopathological features typically include hyperkeratosis, hypergranulosis, and acanthosis, as observed in our case [4].

Treatment options for transgradient Mal De Meleda are limited and mainly aimed at symptom management [12]. Oral retinoids, such as acitretin, are considered the mainstay of therapy due to their ability to reduce hyperkeratosis and improve symptoms [10]. Topical therapies, including urea-based moisturizers, can provide adjunctive relief by softening thickened skin and enhancing its exfoliation. However, long-term management remains challenging, and recurrence is standard following discontinuation of therapy [4].

Our case highlights the importance of early recognition and appropriate management of rare variants of PPK. Despite the challenges posed by its rarity, prompt diagnosis and initiation of therapy can lead to significant improvement in symptoms and quality of life for affected individuals. Further research is warranted to elucidate the pathogenesis of transgradient Mal De Meleda and explore novel therapeutic strategies to address its long-term management needs.

## Conclusions

In conclusion, the presented case underscores the significance of recognizing and addressing rare variants of PPK, such as the transgradient type of Mal De Meleda. Despite its rarity, prompt diagnosis facilitated by clinical suspicion and histopathological examination, coupled with appropriate management, notably with oral retinoids like acitretin and adjunctive topical therapies, can significantly improve symptoms and enhance the quality of life for affected individuals. However, long-term management remains challenging, necessitating further research to elucidate the underlying pathogenesis and explore novel therapeutic avenues. Through continued efforts to understand and manage such rare dermatological conditions, clinicians can optimize patient outcomes and alleviate the burden imposed by these debilitating disorders.
